# Modified single innominate artery cannulation with low flow cardiopulmonary bypass during repair of interrupted aortic arch

**DOI:** 10.1186/1749-8090-8-S1-O240

**Published:** 2013-09-11

**Authors:** Q Chen, M Caputo, S Stoica, G Stuart, A Wolf, A Parry

**Affiliations:** 1 Cardiac Surgery, Bristol Royal Hospital for Children, Bristol, UK; 2 Cardiology, Bristol Royal Hospital for Children, Bristol, UK; 3 Cardiac Anaesthesia, Bristol Royal Hospital for Children, Bristol, UK

## Background

Conventional techniques for repairing interrupted aortic arch (IAA) using deep hypothermic circulatory arrest (DHCA) is known to be associated with the risk of postoperative neurological injury. We have used alternative technique with direct innominate artery cannulation for continuous cerebral perfusion, without DHCA, during repair of IAA in neonates regardless of patient’s weight.

## Methods

Between September 1999 and July 2011, 32 consecutive children with IAA (13 type A, 19 type B) underwent repair using continuous, hypothermic (18 °C) low flow CPB without circulatory arrest. Associated cardiac lesions were Truncus Arteriosus (4), VSD (22), DORV (2), aortopulmonary window (4). Associated cardiac lesions were corrected in all except the DORV which were banded.

## Results

Age at time of surgery was 7 days (4-120 days) and weight 3.1 kg (2.1 to 5.8 kg). Selective cerebral perfusion was maintained in all patients throughout aortic reconstruction. During the period of selective cerebral perfusion, pump flow rate was maintained at 30 mls/kg/min. Aortic cross clamp time, low-flow, and total CPB time were 66 (42-114), 29 (18-41) and 109 (83-217) minutes, respectively. There were no deaths or neurological injury in this series. Postoperative ventilation time, and length of ICU and hospital stay were 3 (2-14), 5 (3-21), and 13 (6-27) days, respectively. Follow-up, complete at 48 months (21-156), revealed no late neurologic sequelae nor innominate artery complications. There were three late re-stenosis of the aortic arch requiring balloon dilatation in 2 and surgical repair in 1.

## Conclusions

Direct innominate arterial cannulation with continuous selective cerebral perfusion can be safely applied during repair of IAA even with low birth weight neonates. It is technical simple and associated with excellent clinical outcomes.

